# Effect on body weight and composition in overweight/obese Australian adults over 12 months consumption of two different types of fibre supplementation in a randomized trial

**DOI:** 10.1186/s12986-016-0141-7

**Published:** 2016-11-17

**Authors:** Sebely Pal, Suleen Ho, Roland J. Gahler, Simon Wood

**Affiliations:** 1 School of Public Health, Curtin University, GPO Box U1987, Perth 6845 WA, Australia; 2 Factors Group Research, Burnaby, Canada; 3 University of British Columbia, Vancouver, Canada

**Keywords:** Obesity, Fibre, PGX, Psyllium, Body composition

## Abstract

**Background/Objectives:**

Higher fibre intakes are associated with risk reduction for chronic diseases. However, many people find difficulty in consuming sufficient fibre through their diet. Supplements may be an effective alternative. We aimed to investigate the effects of PolyGlycopleX® (PGX®), a proprietary polysaccharide complex and a proprietary Psyllium product (PgxSyl™) (PSY) on diet, body weight and composition in overweight and obese individuals.

**Subjects/Methods:**

This was a double-blind 52 weeks study with 159 people randomized to 3 groups: control (rice flour); PGX (PGX) and proprietary psyllium (PSY). Participants did not change any of their usual habits or diet except they consumed 5 g of supplement taken with a total of 500 ml of water 5–10 min before meals.

**Results:**

Weight was significantly lower in the PGX group compared to control at 3 (−1.6 kg [0.57, 2.67, *p* = 0.003]), 6 (−2.6 kg [1.01, 4.13, *p* = 0.001]) and 12 months (−2.6 kg [0.59, 4.64, *p* = 0.012]) and in the PSY group compared to control group at 3 (−1.1 kg [0.07, 2.12, *p* = 0.037]) and 6 months (−2.4 kg [0.95, 3.93, *p* = 0.002]). This was a difference of − 2.8% for the PGX group and − 1.5% for the PSY group compared to control after 12 months supplementation. Body Fat was significantly lower in PGX compared to control at 6 (−1.8 kg [0.63, 2.95, *p* = 0.003]) and 12 months (−1.9 kg [0.43, 3.36, *p* = 0.012]) and in PSY compared to control at 6 (−1.9 kg [0.84, 3.04, *p* = 0.001]) and 12 months (−1.4 kg [0.08, 2.71, *p* = 0.038]).

**Conclusions:**

PGX was better than PSY at maintaining dietary changes and weight loss over the 12 month intervention period, with no change to exercise. A simple strategy of PGX supplementation may offer an effective solution to long-term weight-loss and then management without the need for other nutrient modification.

**Trial registration:**

ANZCTR: ACTRN12611000415909. Registered 20 April 2011

## Background

Dietary fibre recommendations for adults in Australia, Canada and the USA are 25–30 g/d to be consumed from fibre-rich foods [1]. However it is estimated that these countries’ adults consume approximately 15–25 g of dietary fibre/d [2, 3]. Epidemiological and cohort studies have consistently revealed that higher fibre intakes are correlated with lower body weight, body mass index (BMI), waist circumference [4, 5], improved lipid profiles [2, 6–14], glycaemia and insulinaemia [15]; indicating the benefits and risk reduction for metabolic syndrome, cardiovascular disease and type 2 diabetes.

Although the benefits of fibre are well known, people, in general, find it difficult to eat the required amounts of fibre by increasing fruit and vegetable intake [16]. Therefore, fibre supplements can provide an easy, cost effective method for increasing fibre intake without the need for other major nutrient modifications.

### PolyGlycopleX® (PGX®)

PolyGlycopleX (PGX) is a novel, highly viscous functional non-starch polysaccharide complex, with developing viscosity, manufactured by a proprietary process (EnviroSimplex®) from konjac (glucomannan), sodium alginate and xanthan gum. Adding 2.5–5 g of PGX to a meal is highly effective in reducing postprandial glycaemia, lowering the glycaemic index of food [17] and modifying satiety hormones in healthy adults [18].

### Psyllium fibre

Psyllium is one of the most widely used fibre supplements in Australia because it is reasonably cheap, is available in several flavours and sold as powdered drink mixes, capsules or wafers. Psyllium has advantages over other types of soluble fibre because it is less readily fermented and therefore causes less flatulence and abdominal bloating [19]. Psyllium is a soluble fibre and has been evaluated in various human studies for beneficial effects on glucose and insulin homeostasis, lipids and lipoprotein, body weight, body composition and appetite [20–28]. Therefore PSY was chosen as a positive control to examine whether PGX would be as good as or better than PSY. Psyllium intake was reviewed (2012) for its effect on metabolic syndrome [29]. The authors concluded, “Collectively, research to date does support the notion that the consumption of psyllium may provide benefits to many components of the metabolic syndrome.” Psyllium fibre seems to improve body weight in animals [30] but human studies remain controversial, with most showing no improvement on body weight and body composition after psyllium consumption [21, 24–26].

PGX is the most viscous soluble fibre, 3–5 times more viscous than any known individual polysaccharide [31]. Although it is considered natural, it is a blend of 3 different fibres: konjac (glucomannan), sodium alginate and xanthan gum. Psyllium has a similar physical appearance as PGX in its powder form but has a lower viscosity.

Both PGX and psyllium are viscous fibers which absorb large amounts of water and form gels that increase feelings of fullness, [32] this may cause people to consume less food. The thickening of gut contents decreases intestinal passage rates, prolongs nutrient absorption and hence causes satiety [33], thus PGX with a greater viscosity has a greater effect on satiety and reduced food intake [32]. Given the greater viscosity, it was hypothesized that PGX would have better metabolic outcomes in this clinical trial.

Randomized, blinded, placebo controlled clinical trials are required to verify whether PGX® can be used for long term weight loss programs. Therefore the aim of this study was to investigate the effect of PGX on body weight and composition when compared to a proprietary Psyllium (PgxSyl™) product and a placebo. Given the effect of PGX on postprandial glycemia/insulinemia and its considerably higher viscosity, we hypothesize that PGX will demonstrate a greater improvement of body weight loss and composition than the psyllium product or placebo in individuals with overweight and obesity.

## Methods

### Subjects

Individuals with overweight and obesity (BMI 25–47 kg/m^2^) were recruited from the community in Perth, Australia and screened by telephone or online questionnaire. Exclusion criteria included smoking, medication and other agents that may influence lipid metabolism, diabetes mellitus, hypo- and hyperthyroidism, cardiovascular events within the last 6 months, major systemic diseases, gastrointestinal problems, weight fluctuations over the past 6 months, and participation in any other clinical trials within the last 6 months.

### Study design

This was a randomized, double-blind, parallel design study over a 52 weeks period, with recruitment and the intervention conducted from February 2012 to September 2013. Participants were equally randomized by the trial sponsors using a Web site [http://www.randomization.com/] to one of three groups (3 randomly permuted blocks): the control group who consumed the placebo with their usual diet; the psyllium group (PSY) who consumed a psyllium supplement with their usual diet and a PGX group (PGX) who consumed a PGX supplement with their usual diet. The supplementation (InovoBiologic Inc., Calgary, Canada) consisted of either 5 g of psyllium or 5 g of PGX. Placebo consisted of 5 g rice flour, an appropriate placebo due to its low energy and fibre content but similarity in texture and appearance to the psyllium and PGX. All supplements were artificially sweetened (aspartame), naturally flavoured (citric acid and orange) and coloured (sunset yellow FCF). Participants were instructed to take supplements mixed with a minimum of 250 mL water followed by an additional 250 mL water (500 mL total), three times daily, 5–10 min before meals. Packages were identical and were only marked by the participant ID (sequentially numbered), with the group allocation only known to the trial sponsors to ensure blinding. All identifiable information from participants was coded to ensure privacy.

The subjects attended a briefing session on how to consume the supplement, complete food records and comply with the study protocol as previously reported [34]. All participants were asked to maintain their usual dietary intake and physical activity for the duration of the study. To monitor compliance, participants completed a diary to record their supplement consumption and asked to return the empty and non-empty sachets of the supplements at visits.

### Anthropometry and body composition

Measures of weight, height, waist and hip circumference were undertaken at baseline, 3, 6 and 12 months. Weight (HBF-514, Omron, Kyoto, Japan) was recorded in light clothing without shoes and height was measured using a stadiometer. Waist circumference was measured in the standing position at the narrowest area between the lateral lower rib and the iliac crest. Hip measurement was taken at the largest circumference of the lower abdomen. Total body fat, lean mass, android fat and gynoid fat were assessed by whole body dual-energy X-ray absorptiometry (DXA; Lunar Prodigy, Lunar, Madison WI, USA) at baseline, 6 and 12 months. Android fat (fat around the abdomen) and gynoid fat (fat around the hips) regions were automatically obtained by the GE Lunar Prodigy software. Body fat distribution varies by gender as women tend to have greater gynoid fat than men [35].

### Diet and physical activity

Participants completed 3-day food and drink diaries at baseline, 3, 6 and 12 months to monitor for changes in food intake. Data were analysed with Foodworks 7 Professional (Xyris Software, Australia). Participants also completed the International Physical Activity Questionnaire (short version) at the same time points to monitor physical activity levels.

### Statistical analysis

A sample size of 24 subjects/group was predicted to provide sufficient power (80%) to detect a 3% difference in weight within a group. Calculations were based on a mean weight of 80 kg and a standard deviation of 5% within a group on all eligible subjects. We recruited 53 subjects/group to accommodate for 50% dropouts. Statistical analysis was undertaken using SPSS 22 for Windows (SPSS Inc., Chicago, IL, USA). Data were expressed as mean (± SEM) and assessed for normality. Baseline differences between groups was analysed with one-way ANOVA. Changes from baseline within a group were analysed with paired *T*-test. The data were analysed using General Linear Models with baseline value covariates. If significant between groups effects were present, post hoc comparisons between the treatment groups was made using the Least Significant Difference (LSD) method. Statistical significance was considered at *p* <0.05. There were no significant differences between genders when sex was used as a factor in the analysis of variance for each parameter reported, i.e. diet, physical activity, body weight and body composition at each time point.

### Patient involvement

No patients were involved in the design, recruitment to or conduct of the study or in the development of the research question and outcome measures. Individual results were sent to participants. The burden of the intervention on participants was not assessed.

## Results

### Participants

**Fig. 1:**
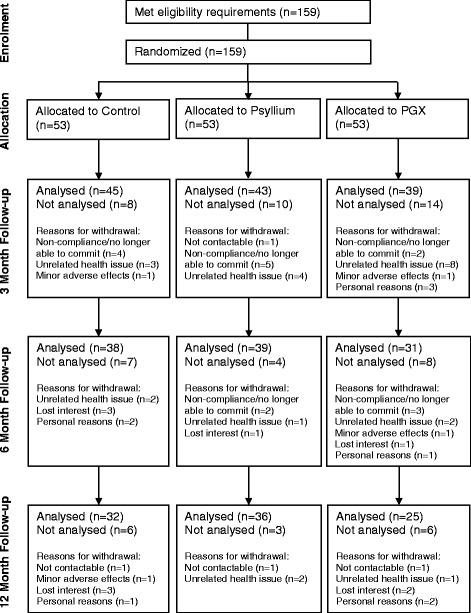
Participant flow diagram

The 159 participants (19 to 68 y) who met the eligibility criteria were randomized to one of three groups (Control, PGX, PSY) by assignment of an ID number from 001 to 159 and the corresponding numbered supplement. Participant flow through the study can be seen in Fig. 1. 127 participants (54 male, 73 female) completed at least 3 months of the study and were included in the analysis (45 in Control [24 male], 39 in PGX [15 male] and 43 in PSY [15 male]).

### Baseline characteristics

There were no significant differences at baseline between groups for major characteristics, energy intake, macronutrients or fibre intake (Table 1).

**Table 1:** Baseline characteristics

	Control (*n* = 45)	Psyllium (*n* = 43)	PGX (*n* = 39)
Gender (M/F)	24/21	15/28	15/24
Age (y)	49.8 ± 1.8	49.9 ± 1.7	47.9 ± 1.9
Height (cm)	171.7 ± 1.5	169.2 ± 1.6	169.8 ± 1.7
Weight (kg)	94.7 ± 2.5	91.2 ± 2.2	96.2 ± 2.9
BMI (kg/m^2^)	32 ± 0.6	31.7 ± 0.5	33.2 ± 0.7
Waist (cm)	103.1 ± 1.6	101.2 ± 1.5	106 ± 2
Hip (cm)	112.9 ± 1.3	114.6 ± 1.3	115.7 ± 1.5
Waist Hip Ratio	0.91 ± 0.01	0.88 ± 0.01	0.92 ± 0.02
	Control (*n* = 44)	Psyllium (*n* = 43)	PGX (*n* = 38)^a^
Fat (kg)	37.3 ± 1.4	38.6 ± 1.2	38.3 ± 1.3
Fat %	40.2 ± 1.2	40.89 ± 1.1	41.1 ± 1.2
Android Fat (kg)	3.8 ± 0.2	3.5 ± 0.2	3.9 ± 0.2
Gynoid Fat (kg)	6.1 ± 0.3	6.3 ± 0.2	6.2 ± 0.3
Lean (kg)	53 ± 1.9	50.4 ± 1.7	52.8 ± 2.2
Lean %	56.6 ± 1.2	55.8 ± 1	55.7 ± 1.2
	Control (*n* = 44)	Psyllium (*n* = 43)	PGX (*n* = 39)
Energy (kJ/d)	8636.4 ± 294.4	8859.4 ± 318.4	8783.3 ± 261.3
Carbohydrate (g/d)	209.8 ± 8.8	212 ± 8.9	209.8 ± 8.6
Fat (g/day)	80.2 ± 4.2	81.2 ± 4.5	82.8 ± 3.3
Protein (g/d)	101.7 ± 4	104 ± 4.7	101.6 ± 3.9
Fibre (g/d)	22.6 ± 1.2	23.9 ± 1.3	21.4 ± 1.2
	Control (*n* = 40)	Psyllium (*n* = 41)	PGX (*n* = 35)
Physical Activity^b^ (MET/min/w)	3058 ± 591	3133 ± 453	2135 ± 432

Values are mean ± SEM. PGX (PolyGlycopleX) MET (Metabolic Equivalent of Task). ^a^One participant in the PGX group was unable to have the DXA scan. ^b^Physical activity logs missing (*n* = 10). *P* values were not significant. 4.184 kJ = 1 kCal

### Diet

The dietary analysis can be seen in Table 2. We observed significant decreases in energy intake in the PGX group at 3 months (*p* = 0.001), 6 months (*p* = 0.009) and 12 months compared to baseline (*p* = 0.012). Energy intake was significantly lower in the PSY group at 3 months (*p* = 0.000), 6 months (*p* = 0.000) and 12 months (*p* = 0.001) compared to baseline. When examining differences between groups, energy intake was significantly lower compared to control at 3 months in the PGX® (−16.2%, *p* = 0.000) and PSY (−19.3%, *p* = 0.000) groups, at 6 months in PGX (−15.3%, *p* = 0.002) and PSY (−14.4%, *p* = 0.002) groups compared to control and at 12 months in the PGX group (−11%, *p* = 0.049) compared to control.

**Table 2:** Dietary intake during 12 months of fibre supplementation

Variable		3 months	Mean change	*n*	*P*	6 months	Mean change	*n*	*P*	12 months	Mean change	*n*	*P*
Energy (kJ/d)	CTR	9013.1 ± 223.6^a^	115.5	39	NS	8803.3 ± 282.9^a^	32	34	NS	8218 ± 295.4^a^	−529.7	31	NS
	PSY	7272.3 ± 218^b^	−1617.3	41	0.000	7539.2 ± 278.8^b^	−1229.2	35	0.000	7657.1 ± 277.9^ab^	−1202	35	0.001
	PGX	7556.3 ± 243.1^b^	−1090.9	33	0.001	7453.7 ± 323.5^b^	−1080.7	26	0.009	7315.3 ± 342.4^b^	−1226.4	23	0.012
CHO (g/d)	CTR	212.6 ± 7.7^a^	−1.6	39	NS	213.4 ± 10^a^	3.4	34	NS	200.5 ± 8.4	−10.3	31	NS
	PSY	181 ± 7.5^b^	−31.8	41	0.002	164.7 ± 9.8^b^	−39.9	35	0.002	183.9 ± 7.9	−26	35	0.015
	PGX	175.3 ± 8.4^b^	−31.5	33	0.003	177.2 ± 11.4^b^	−22.1	26	0.032	176.3 ± 9.7	−25.6	23	0.012
Fat (g/d)	CTR	83.8 ± 3.2^a^	−0.5	39	NS	82.3 ± 4.3^a^	−0.6	34	NS	73 ± 4	−9.5	31	NS
	PSY	66.9 ± 3.1^b^	−15.6	41	0.000	72.9 ± 4.2^ab^	−8.6	35	NS	73 ± 3.8	−9.6	35	NS
	PGX	72.2 ± 3.4^b^	−9	33	NS	69.1 ± 4.9^b^	−10.5	26	0.047	66 ± 4.7	−15.2	23	0.020
Protein (g/d)	CTR	109.9 ± 3.7^a^	6.4	39	NS	105.2 ± 4.1^a^	3.1	34	NS	99.4 ± 4.7	−2.1	31	NS
	PSY	85.9 ± 3.6^b^	−17.6	41	0.000	96.7 ± 4^ab^	−6.7	35	NS	88 ± 4.4	−15.3	35	0.003
	PGX	94.4 ± 4.1^b^	−6.5	33	NS	85.7 ± 4.7^b^	−15.7	26	0.004	89.2 ± 5.4	−10.2	23	NS
Fibre from diet (g/d)	CTR	24.2 ± 1.2	0.8	39	NS	22.1 ± 1^a^	−0.4	34	NS	21.6 ± 1.3	−1	31	NS
	PSY	21.4 ± 1.2	−2.3	41	NS	20.1 ± 0.9^ab^	−2.9	35	0.005	21.6 ± 1.3	−2.4	35	NS
	PGX	21.6 ± 1.3	−0.6	33	NS	19.1 ± 1.1^b^	−2.2	26	NS	21.1 ± 1.6	−1	23	NS
Total Fibre (g/d)	CTR	24.2 ± 1.2^a^	0.8	39	NS	22.1 ± 1^a^	−0.4	34	NS	21.6 ± 1.3^a^	−1	31	NS
	PSY	36.4 ± 1.2^b^	12.7	41	0.000	35.1 ± 0.9^b^	12.1	35	0.000	36.6 ± 1.3^b^	12.6	35	0.000
	PGX	36.6 ± 1.3^b^	14.2	33	0.000	34.1 ± 1.1^b^	12.5	26	0.000	36.1 ± 1.6^b^	13.8	23	0.000

Values are mean ± SEM with baseline as a covariate. Mean change from baseline. *P* values are within group differences compared to baseline. Different letters in superscript represent significant differences between groups *p* < 0.05. CHO (carbohydrate), CTR (control), PGX (PolyGlycopleX), PSY (psyllium). Dietary intake data missing at baseline or 3 months (*n* = 14). 4.184 kJ = 1 kCal

Carbohydrate intake was significantly lower compared to control at 3 months in the PGX (*p* = 0.001) and PSY (*p* = 0.004) groups and at 6 months in PGX (*p* = 0.019) and PSY (*p* = 0.001) groups compared to control. Fat intake was significantly lower compared to control at 3 months in the PGX (*p* = 0.014) and PSY (*p* = 0.000) groups and at 6 months in the PGX group (*p* = 0.045) compared to control. Protein intake was significantly lower compared to control at 3 months in the PGX (*p* = 0.006) and PSY (*p* = 0.000) groups and at 6 months in the PGX® group (*p* = 0.002) compared to control. Compared to baseline, carbohydrate intake was significantly lower for both PGX and PSY groups at all time points. Fat and protein intakes were inconsistent during the 12 month study period.

Dietary fibre intake from food was significantly lower compared to control at 6 months in the PGX (*p* = 0.045) group. Fibre intake from food was significantly lower in the PSY group at 6 months (*p* = 0.005) compared to baseline. Total fibre intake in the PGX and PSY intervention groups was 15 g/day higher than fibre intake from food, as participants were consuming three 5 g fibre supplements each day. This was a significant increase compared to baseline and compared to the control group for both PGX and PSY groups.

### Physical activity

Physical activity levels did not significantly change from baseline within any groups and there were no significant differences between groups at any time point (Table 3).

**Table 3:** Physical activity during 12 months of fibre supplementation

	3 months	Mean change	*n*	6 months	Mean change	*n*	12 months	Mean change	*n*
CTR	2802.4 ± 449.3	−130.5	29	2868.2 ± 454.7	−141.2	32	3294.5 ± 525.6	415.4	30
PSY	2933 ± 443.6	620.8	30	3009.3 ± 433.2	187	35	2879.1 ± 497.8	609.6	33
PGX	2181.7 ± 470.6	751.3	27	2681.1 ± 495.5	328.2	27	2684.9 ± 619.1	194.3	22

Values are mean kJ/day ± SEM with baseline as a covariate. Mean change from baseline. Physical activity data missing at either baseline and/or at follow up time points (*n* = 67). Gender ratio was unaffected at 2 males:3 females. *P* values were not significant. 4.184 kJ = 1 kCal

### Body weight

**Fig. 2:**
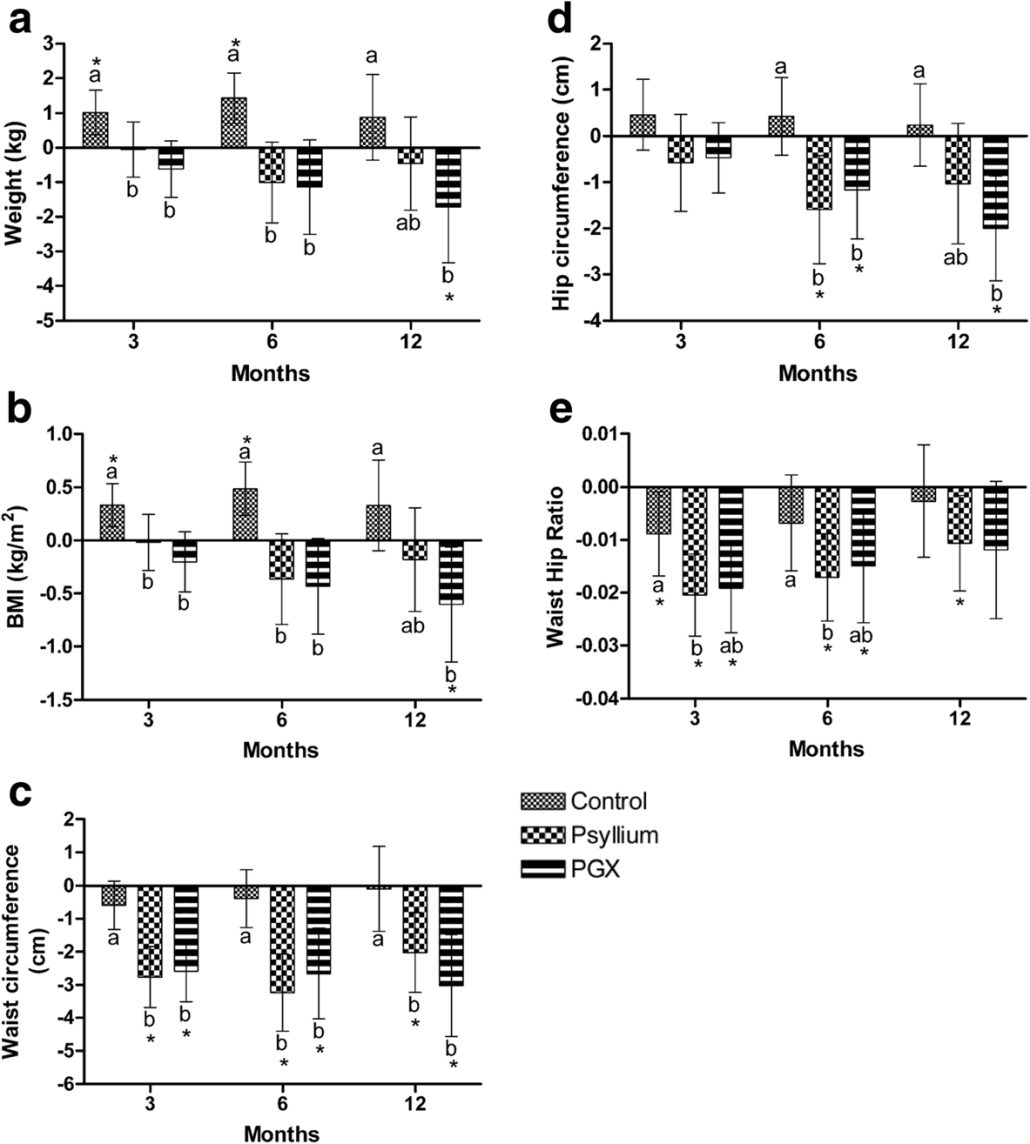
Change in body weight and waist during12 months of fibre supplementation **a** Weight, **b** BMI, **c** Waist, **d** Hip, **e** WHR. Values are changes in the parameters over the 12 months and are mean ± 95% CI error bars with baseline as a covariate. *indicates within group differences compared to baseline. Different letters represent significant differences between groups *p* < 0.05. 3 months *n* = 45 CTR, 43 PSY, 40 PGX, 6 months *n* = 38 CTR, 39 PSY, 32 PGX, 12 months *n* = 32 CTR, 36 PSY, 26 PGX. CTR Control, PSY Psyllium, PGX (PolyGlycopleX)

Weight and BMI were significantly lower in the PGX group at 12 months compared to baseline (*p* < 0.05), Fig. 2a shows change in body weight Fig. 2b shows change in BMI. Weight was significantly lower compared to control at 3 months in the PGX (−1.7%, *p* = 0.007) and PSY (−1.2%, *p* = 0.037) groups, at 6 months in PGX (−2.7%, *p* = 0.001) and PSY (−2.6%, *p* = 0.002) groups compared to control and at 12 months only in the PGX group (−2.8%, *p* = 0.012) compared to control. BMI was significantly lower compared to control at 3 months in the PGX (*p* = 0.004) and PSY (*p* = 0.042) groups, at 6 months in PGX (*p* = 0.001) and PSY (*p* = 0.001) groups compared to control and at 12 months only in the PGX group (*p* = 0.010) compared to control.

Waist circumference was significantly lower (*p* < 0.01) in the PGX and PSY groups at 3 months, 6 months and 12 months compared to baseline, Fig. 2c. Waist was significantly lower compared to control at 3, 6 and 12 months in the PGX and PSY groups (*p* < 0.01) compared to control.

Hip was significantly lower in the PGX group at 6 and 12 months (*p* < 0.05) compared to baseline. Hip was significantly lower in the PSY group 6 months (*p* = 0.009) compared to baseline. Hip was significantly lower compared to control at 6 months in PGX (*p* = 0.034) and PSY (*p* = 0.004) groups compared to control and at 12 months only in the PGX group (*p* = 0.008) compared to control, Fig. 2d.

WHR was significantly lower in the PGX group at 3 and 6 months compared to baseline and in the PSY group at 3, 6 and 12 monthsnths compared to baseline (*p* < 0.05). WHR was significantly lower in the PSY group compared to control at 3 and 6 months (*p* < 0.05), Fig. 2e.

### DXA Body fat

**Fig. 3:**
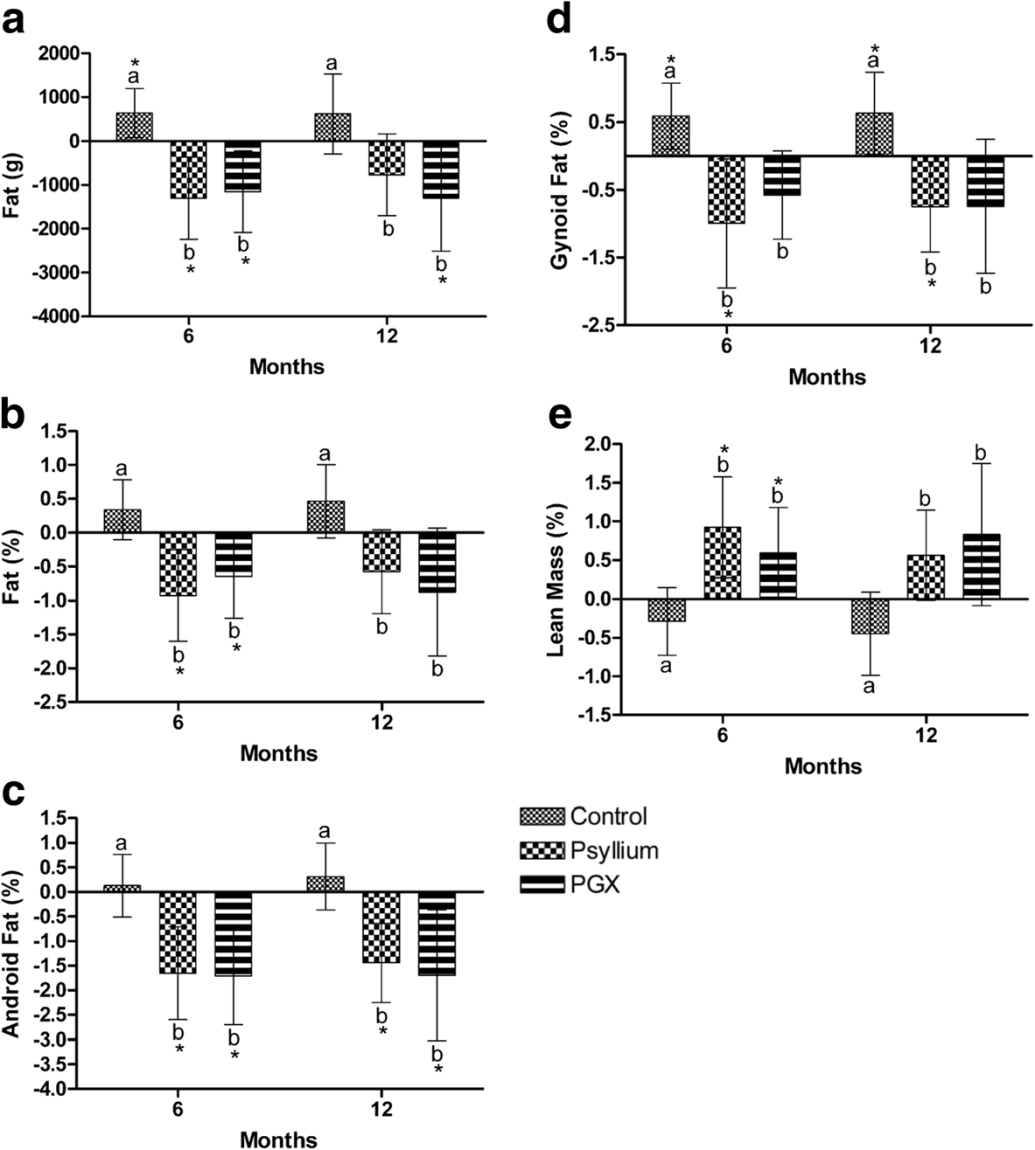
Change in body composition during12 months of fibre supplementation **a** Fat, **b** Fat percentage, **c** Android Fat, **d** Gynoid Fat, **e** Lean Mass. Values are changes in the parameters over the 12 months and are mean ± 95% CI error bars with baseline as a covariate. *indicates within group differences compared to baseline. Different letters represent significant differences between groups *p* < 0.05. 6 months *n* = 37 CTR, 37 PSY, 31 PGX, 12 months *n* = 32 CTR, 35 PSY, 26 PGX. CTR Control, PSY Psyllium, PGX (PolyGlycopleX)

The amount of change of body fat was significantly lower in the PSY and PGX groups at 6 months and in PGX group at 12 mo (*p* < 0.05) compared to baseline. Body fat was significantly lower compared to control at 6 months in the PSY (*p* = 0.001) and PGX® (*p* = 0.003) groups and at 12 months in the PSY (*p* = 0.038) and PGX groups (*p* = 0.012) compared to control, Fig. 3a. Body fat % was significantly lower in the PGX and PSY groups at 6 months compared to baseline (*p* < 0.05). Body fat % was significantly lower compared to control at 6 months in the PGX (*p* = 0.020) and PSY (*p* = 0.002) groups and at 12 months in the PGX (*p* = 0.008) and PSY groups (*p* = 0.018) compared to control, Fig. 3b.

Android fat was significantly lower in the PGX and PSY groups at 6 and 12 months (*p* < 0.05) compared to baseline. Android fat was significantly lower compared to control at 6 months in the PGX (*p* = 0.004) and PSY (*p* = 0.003) groups and at 12 months in the PGX (*p* = 0.003) and PSY groups (*p* = 0.005) compared to control, Fig. 3c. Gynoid fat was significantly lower in the PSY group at 6 and 12 months (*p* < 0.05) compared to baseline. Gynoid fat was significantly lower compared to control at 6 months in the PGX® (*p* = 0.028) and PSY (*p* = 0.002) groups and at 12 months in the PGX (*p* = 0.010) and PSY groups (*p* = 0.003) compared to control, Fig. 3d.

Lean mass % was significantly higher in the PGX and PSY groups at 6 months (*p* < 0.05) compared to baseline. Lean mass % was significantly higher compared to control at 6 months in the PGX (*p* = 0.035) and PSY (*p* = 0.002) groups and at 12 months in the PGX (*p* = 0.008) and PSY groups (*p* = 0.017) compared to control, Fig. 3e.

### Adverse events

Minor adverse events were gastrointestinal related (e.g. flatulence, diarrhoea) with four participants withdrawing from the study, two in the PGX group and two in the control group. The PSY supplement was better tolerated and participants did not report any adverse effects.

## Discussion

This study investigated the effects of daily consumption of 15 g of PGX or PSY compared to control (rice flour) for 1 year. Both the PGX and PSY groups demonstrated significant reductions in energy and macronutrient intake as well as significant weight loss and improvements to body composition at 52 weeks. At12 months, energy intake and weight was significantly lower in the PGX group compared to control but was not significantly lower in the PSY group compared to control. Fat and protein intake was significantly lower in the PGX group at 3 and 6 months compared to control whereas fat and protein intake was only significantly lower in the PSY group at 3 months compared to control. In this regard, the PGX group performed better than the PSY group and was better at maintaining dietary changes and weight loss over the 12 month intervention period.

We observed significant decreases in energy and macronutrient intake within the two intervention groups at 3, 6 and 12 months compared to baseline. Although participants were instructed not to intentionally change their diet, taking the supplements daily before each meal resulted in a decrease in their overall food intake (Table 1). The short term consumption of PGX has been previously shown to reduce hunger and food consumption in healthy weight adolescents [32] and increase satiety or feelings of fullness in normal weight adults [36] and women who were overweight/obese [37]. This is thought to be through its effects on appetite-regulating peptides glucagon-like peptide-1 and peptide YY [38] and/or due to its gel forming property that increases stomach fullness and delays gastric emptying [36].

In previous retrospective observational clinical trials, overweight participants lost significant weight, fat and decreased waist circumference compared to baseline after taking PGX for 14 weeks in addition to healthy lifestyle changes [39]. In addition, overweight subjects taking a PGX meal replacement and a PGX supplement for 12 weeks also experienced significant decreases in weight, waist and hip measurements compared to baseline [40]. This is in agreement with the current study and at the same daily dose of 15 g of PGX. However, in contrast to these two previous trials, we were able to demonstrate significant changes in weight, waist and body fat in the PGX group compared to the control group for 52 weeks and without prescribing any other lifestyle changes.

Psyllium is widely used as a fibre supplement as it is readily available and well tolerated, however findings on the effect on metabolic syndrome risk factors have been inconclusive [29]. De Bock et al. [41], conducted a 6 weeks intervention crossover study with 6 g/day of psyllium supplementation in healthy adolescents and did not observe any significant change in dietary intake, weight or body fat percentage. In a study by Pal et al. [20] psyllium supplementation (3.4 g psyllium per 12 g dose of Metamucil® with 250 ml of water) caused significant decreases in body weight, BMI and body fat compared to the control group after 12 weeks but no differences in waist circumference. In the current study, the PSY group significantly decreased body weight, BMI and body fat compared to the control group but we also observed significant decreases in energy intake and waist circumference. However, in contrast to the studies by de Bock et al. [41] and Pal et al. [20], our supplement was 5 g per dose taken with 500 ml of water. The size of the dose or the amount of water consumed may be an important factor when taking a fibre supplement and an adequate amount needs to be taken to be effective.

The weight loss and changes to body composition observed may be due to the changes in dietary intake as well as the possible effect of PGX on slowing gastric emptying and absorption of nutrients in the small intestine [42]. In the current study, energy intake demonstrated significant positive relationships (*p* < 0.05) with body weight (*r* = 0.360), abdominal fat (*r* = 0.223) and lean mass (*r* = 0.378) across all participants. A PGX containing diet has also been shown to lower food intake and slow weight gain in rats compared to cellulose or inulin containing chow [43]. PSY has also been shown to increase fullness [27] but has a lower viscosity than PGX [39].

One of the strengths of this study was the duration; a 12 month intervention period. Comparable studies have only been conducted for 14 weeks. This allowed us to investigate the long term effects of the supplements, especially on weight maintenance. This was a double-blinded randomized study and supplements were packed in identical foil sachets; however, due to the different characteristics of the supplements, participants may have been able to guess if they were taking a supplement or the control. The majority of participants were female despite a higher prevalence of overweight and obesity in males in the Australian setting [44], so results may not be generalizable to males or populations in other countries. Other limitations include our reliance on participant honesty and accuracy when completing food diaries and reporting of supplement consumption. Care must be taken when drawing conclusions from self-reported data, however more reliable methods of measuring food intake with low participant burden are lacking. The intervention was not combined with any other lifestyle modification advice, thus it would be simple for consumers to incorporate into their lifestyle or there could be added benefits if the supplements were combined with healthy lifestyle advice.

## Conclusions

The effects of PGX on appetite-regulating peptides may also play a role in reducing dietary intake. Taking these supplements before meals was a relatively easy task for people to incorporate into their daily routine and would be a simple intervention to implement. We observed similar results between PGX and PSY supplements but when compared to the control group, the PGX supplement was superior in terms of increased weight loss and decreased energy intake. Therefore, regular consumption of a PolyGlycopleX® or the proprietary psyllium supplement is a simple and effective method to reduce body weight and body fat in people with overweight or obesity. Further work on these two proprietary products should be undertaken to investigate these initial findings in other population groups and in other formats, for example, a PGX softgel or a psyllium softgel.

## Abbreviations

BMI: Body mass index
CHO: Carbohydrate
CTR: Control
DXA: Dual-energy X-ray absorptiometry
LSD: Least significant difference
PGX: PolyGlycopleX
PSY: Psyllium
WHR: Waist to hip ratio
